# Oxidase Reactivity of Cu^II^ Bound to *N*-Truncated Aβ Peptides Promoted by Dopamine

**DOI:** 10.3390/ijms22105190

**Published:** 2021-05-14

**Authors:** Chiara Bacchella, Simone Dell’Acqua, Stefania Nicolis, Enrico Monzani, Luigi Casella

**Affiliations:** Dipartimento di Chimica, Università di Pavia, Via Taramelli 12, 27100 Pavia, Italy; chiara.bacchella01@universitadipavia.it (C.B.); simone.dellacqua@unipv.it (S.D.); stefania.nicolis@unipv.it (S.N.); enrico.monzani@unipv.it (E.M.)

**Keywords:** copper, amyloid-β peptides, Alzheimer’s disease, oxidative stress, dopamine, neurodegeneration

## Abstract

The redox chemistry of copper(II) is strongly modulated by the coordination to amyloid-β peptides and by the stability of the resulting complexes. Amino-terminal copper and nickel binding motifs (ATCUN) identified in truncated Aβ sequences starting with Phe4 show very high affinity for copper(II) ions. Herein, we study the oxidase activity of [Cu–Aβ_4−x_] and [Cu–Aβ_1−x_] complexes toward dopamine and other catechols. The results show that the Cu^II^–ATCUN site is not redox-inert; the reduction of the metal is induced by coordination of catechol to the metal and occurs through an inner sphere reaction. The generation of a ternary [Cu^II^–Aβ–catechol] species determines the efficiency of the oxidation, although the reaction rate is ruled by reoxidation of the Cu^I^ complex. In addition to the *N*-terminal coordination site, the two vicinal histidines, His13 and His14, provide a second Cu-binding motif. Catechol oxidation studies together with structural insight from the mixed dinuclear complexes Ni/Cu–Aβ_4−x_ reveal that the His-tandem is able to bind Cu^II^ ions independently of the ATCUN site, but the *N*-terminal metal complexation reduces the conformational mobility of the peptide chain, preventing the binding and oxidative reactivity toward catechol of Cu^II^ bound to the secondary site.

## 1. Introduction

Alzheimer’s disease (AD) is the most common dementia leading to progressive impairment of mental and physical functions and is neuropathologically identified by the presence of insoluble deposits of fibrillary plaques incorporating high amounts of amyloid-β peptide (Aβ) and tau fibrillary tangles [1]. The heterogeneous population of Aβ peptides found in the aggregates is derived from a common precursor protein, APP, through the activity of several aminopeptidases [2]. Besides the more extensively studied Aβ_1–40_ and Aβ_1–42_ peptide fragments, generated by the sequential action of two secretases, namely γ- and β-secretase, other species of *N*-terminal and *C*-terminal truncated Aβ fragments have been extracted from neuronal tissues of healthy subjects as well as those with Down syndrome and Alzheimer’s disorder [3,4]. In particular, the miscellaneous population of amyloid fragments comprises *N*-terminal deletion towards Ala2, Glu3, or Phe4, with Aβ_4−x_ peptide as the dominant species extracted from cortex and hippocampus of AD individuals [5].

As it is widely accepted, the etiopathogenesis of AD is also linked to unusually high levels of redox-active metals, particularly copper, within amyloid deposits, which can generate neurotoxic complexes leading to widespread oxidative stress condition in AD brain [6]. Oxidative-induced inflammation is a common trait that is present as an additional cause of various neuronal pathologies [7,8].

In AD, marked differences in the metal interaction ability have been revealed between the full-length Aβ_1−x_ and the *N*-terminally truncated forms: in particular, Aβ_4−x_ bears a peculiar motif H_2_N–Xaa–Yaa–His, known as amino-terminal copper and nickel binding motif (ATCUN), previously detected in some proteins involved in the storage or transport of copper ions, especially serum albumin. This domain shows very high affinity (K_a_ = 3 × 10^13^ M^−1^ at pH 7.4) for copper(II) [9] that is enabled by the metal coordination of terminal (Phe4) amine, two deprotonated backbone amides (Arg5 and His6), and the His6 imidazole group in a system of fused three-membered (5,5,6) chelate rings [10]. An additional binding site in the Aβ sequence consists of the His-tandem site (His13, His14), which is mainly considered as a preferential binding site for Cu^I^, while exhibiting an affinity for Cu^II^ ions 7 orders of magnitude lower than the ATCUN motif. The simultaneous presence of ATCUN and His-tandem sites in Aβ_4−x_ may suggest a functional role of these peptides in the transport and regulation of copper ions since a similar configuration was found in transmembrane Cu transport protein (CTR1) and in human salivary antimicrobial peptides (AMP), such as histatin-5 (Hst5) [11,12].

Controversial results were reported about the redox behavior of copper(II) bound to the *N*-terminally truncated Aβ_4–x_ forms: while some papers reported complete redox-inertness of Cu^II^ when trapped into the ATCUN motif, others indicate a basal production of reactive oxygen species (ROS) by this center, besides the existence of a secondary Cu^II^–His_2_ site (His13, His14) having a modest redox chemistry [13,14,15]. A recent study suggests that ROS production is not fully silenced by Aβ_4−x_ peptides when copper(I) is present in the medium [16].

The present work aims at clarifying the effective lack of reactivity of Cu^II^ bound at the *N*-terminal site of Aβ_4−x_ peptides, in contrast with the possible redox activation of the *C*-terminal His-tandem site, encouraging the metal redox cycling through the reaction with catechol substrates. Indeed, dopamine (DA) and other catecholamines are important messengers and modulators for neuronal signaling, and thus an elaborate control apparatus tightly regulates their storage and release. The dysfunctions of the DA system and the presence of excessive cytosolic or extraneuronal catecholamines widely contribute to exacerbating AD through several mechanisms. In particular, under oxidative stress conditions characterized by elevated lipid peroxidation, metal accumulation, and antioxidant depletion, catecholamines can be quickly converted to the corresponding *o*-quinones and subsequently to high-molecular-weight oxidative melanic products [17,18]. In addition, DA and its metabolites can easily interact with circulating metal ions, promoting metal neurotoxicity, or give rise to nonselective post-translational modification of peptides and proteins [19,20].

Herein, we compare the oxidative properties of Cu–Aβ_4−x_ complexes with those of the corresponding Cu–Aβ_1−x_ (x = 16 or 28) complexes in the presence of DA and other catechol substrates, assuming the potential occupation of the two copper-binding sites, the ATCUN and the His-tandem sites, within the peptides. We also analyze the differences in the peptide vulnerability toward the oxidative environment.

## 2. Results

### 2.1. Oxidative Reactivity of Cu–Aβ_4−x_ and Cu–Aβ_1−x_ Complexes toward Catechols

The oxidative reactivity of copper bound to Aβ_1−x_ and Aβ_4−x_ peptides toward DA and 4-methylcatechol (MC) was studied by UV-Vis spectroscopy following the generation of dopaminochrome (at 475 nm) and 4-methylquinone (401 nm), respectively. DA oxidation is slower than MC oxidation because of the lower semiquinone/catechol redox potential [21] and leads to the formation of mixtures of insoluble oligomeric products. As observed in a previous study [22], at high catechol concentration, the presence of Aβ_1−x_ peptides increases the rate of copper(II)-mediated oxidation, whereas the redox cycling of copper(II) is disfavored at low substrate concentration.

**Figure 1:**
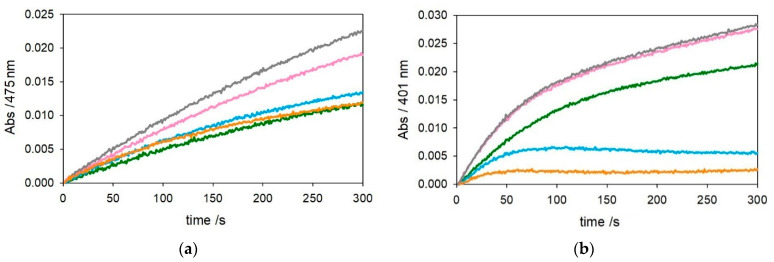
Kinetic profiles of DA (3 mM, panel (**a**)) and MC (3 mM, panel (**b**)) oxidation with time in 50 mM HEPES buffer at pH 7.4 and 20 °C in the presence of Cu^II^ alone (25 µM, green trace) and with 1 equiv. Aβ_1–16_ (25 µM, pink), 2 equiv. Aβ_1–16_ (50 µM, grey), 1 equiv. Aβ_4–16_ (25 µM, light blue), and 2 equiv. Aβ_4–16_ (50 µM, orange).

**Figure 2:**
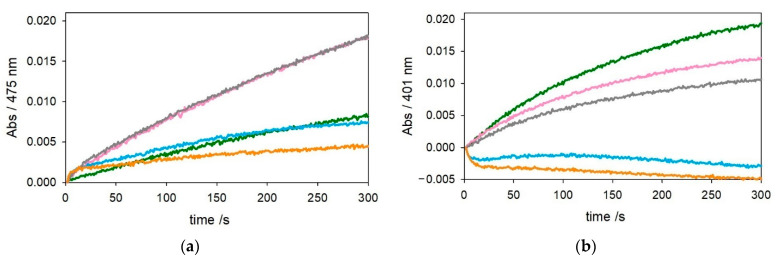
Kinetic profiles of DA (0.3 mM, panel (**a**)) and MC (0.3 mM, panel (**b**)) oxidation with time in 50 mM HEPES buffer at pH 7.4 and 20 °C in the presence of Cu^II^ alone (25 µM, green trace) and with 1 equiv. Aβ_1–16_ (25 µM, pink), 2 equiv. Aβ_1–16_ (50 µM, grey), 1 equiv. Aβ_4–16_ (25 µM, light blue), and 2 equiv. Aβ_4–16_ (50 µM, orange).

The kinetic traces in Figure 1 and Figure 2 show that, as expected, [Cu–Aβ_4–16_] is much less reactive than [Cu–Aβ_1−x_] in the oxidation of DA and MC under both saturating (3 mM—Figure 1) or subsaturating (0.3 mM—Figure 2) conditions. This effect is due to the stabilization of copper(II) trapped by the ATCUN motif, although it is clear from the residual reactivity that [Cu–Aβ_4–16_] is not redox-inert.

We also extended the catechol oxidation experiments to the copper(II) complexes with the longer Aβ_1–28_ and Aβ_4–28_ peptides because they better mimic the behavior of the full-length Aβ_1–40/42_ and Aβ_4–40/42_ peptides but, lacking the highly hydrophobic *C*-terminal tail, their aggregation is much slower [22,23,24,25]. In addition, the studies of copper(II) bound to the ATCUN motif were limited so far to the Aβ_4–16_ peptide [9,26].

**Figure 3:**
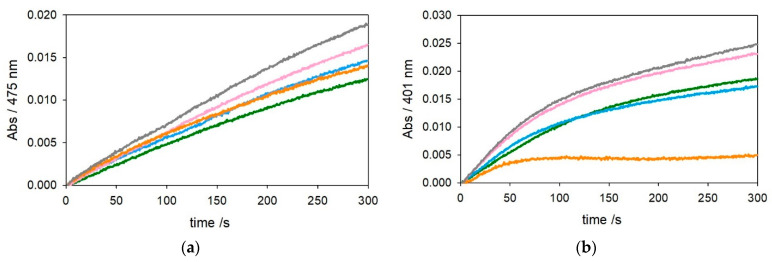
Kinetic profiles of DA (3 mM, panel (**a**)) and MC (3 mM, panel (**b**)) oxidation with time in 50 mM HEPES buffer at pH 7.4 and 20 °C in the presence of Cu^II^ alone (25 µM, green trace) and with 1 equiv. Aβ_1–28_ (25 µM, pink), 2 equiv. Aβ_1–28_ (50 µM, grey), 1 equiv. Aβ_4–28_ (25 µM, light blue), and 2 equiv. Aβ_4–28_ (50 µM, orange).

**Figure 4:**
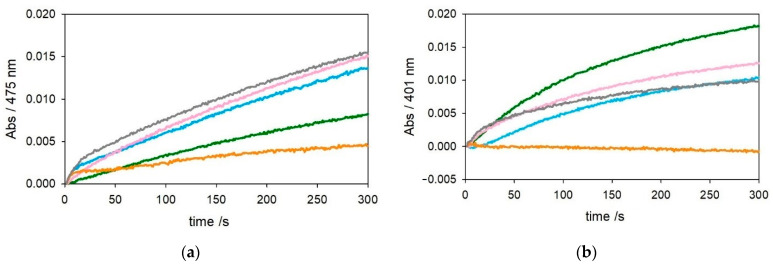
Kinetic profiles of DA (0.3 mM, panel (**a**)) and MC (0.3 mM, panel (**b**)) oxidation with time in 50 mM HEPES buffer at pH 7.4 and 20 °C in the presence of Cu^II^ alone (25 µM, green trace) and with 1 equiv. Aβ_1–28_ (25 µM, pink), 2 equiv. Aβ_1–28_ (50 µM, grey), 1 equiv. Aβ_4–28_ (25 µM, light blue), and 2 equiv. Aβ_4–28_ (50 µM, orange).

The optical traces shown in Figure 3 and Figure 4, referring to oxidation of catechols at 3 mM and 0.3 mM, respectively, show a trend of substrate oxidation rates by [Cu^II^–Aβ_1–28_] and [Cu^II^–Aβ_4–28_] similar to that of their [Cu–Aβ_x−16_] analogs, but the stabilization of Cu^II^ granted by the longer peptide seems to be reduced. On the other hand, the presence of an excess of Aβ_4–28_ (2 equiv.) causes quenching of the reaction, suggesting that a small fraction of peptide may have aggregated during preparation of the experiment in solution. To ensure the presence of monomeric, nonaggregated peptide, we pretreated Aβ_4–28_ with hexafluoro-2-propanol (HFIP) [25]. The kinetic traces in Figure S1 indicate that the presence of only 1.2 equiv. of HFIP-treated Aβ_4–28_ with respect to the metal is already sufficient for quenching of Cu^II^ redox activity. The residual reactivity of copper(II) in the presence of 1 equiv. of Aβ_4–28_ peptide may also indicate that the longer sequence is less efficient in the stabilization of copper(II) compared to Aβ_4–16_.

Another aspect that should be taken into account is the concomitant oxidative modification of the peptide promoted by copper(II) in the presence of oxygen and catechol, as reported in our previous studies [25,27]. HPLC-MS analysis allows the oxidative modifications undergone by Aβ peptides to be identified and quantitated. For Aβ_4–16_, it was not possible to find an experimental setting for reproducible HPLC-MS analysis, but for Aβ_4–28_, extensive oxidative modifications were observed (Figure S2, Tables S1 and S2). Indeed, after 15 min of reaction with each substrate, about 20% Aβ_4–28_ is modified through the insertion of single or multiple oxygen atoms mainly on His13 and His14 (shown as +16, +32, or +48 mass increment), while the covalent attack by catechol/quinone has a minor impact, and negligible levels of fragmentation were obtained in the presence of DA. These modifications are likely to induce the release of free copper, explaining the residual reactivity observed for [Cu^II^–Aβ_4–28_].

To gain additional information regarding the interaction between copper(II), Aβ, and catechol, we monitored the entire visible absorption profile of DA oxidation mediated by [Cu^II^–Aβ_1–16_] and [Cu^II^–Aβ_4–16_] over time. As shown in Figure S3, an absorption band at 300 nm is immediately generated upon the addition of copper(II) to the mixture containing the substrate and the Aβ peptide. This band is attributable to a charge transfer transition from catecholato ligand to copper(II) since it is also observed upon mixing copper(II) and catechol in the absence of peptide (Figure S4). These observations indicate that the substrate binds to copper(II) in [Cu^II^–Aβ_x−16_] complexes and suggest that both Cu^II^–catechol and [Cu^II^–Aβ–catechol] species share the spectral feature at 300 nm. In the case of [Cu–Aβ_1–16_] and free Cu^II^, the band intensifies with the progress of the reaction, with a parallel trend with product formation, while with [Cu–Aβ_4–16_] the band initially present decreases with the progress of the reaction. Moreover, a similar decay of the 300 nm band can be also noticed in the reactions of copper(II) complexes with the longer Aβ_x−28_ fragments in the catechol oxidations (Figure S5). The formation of this ternary [Cu^II^–Aβ–catechol] species is independent of the mixing order, since incubation of metal and peptide before the addition to the substrate solution gave the same absorbance/time profile as when the complex was generated directly in the cell (Figure S6). The lifetime of this [Cu^II^–Aβ–catechol] species is not related to an interaction with dioxygen, as verified by the initial 10 s of the kinetic traces obtained for [Cu–Aβ_4–16_] in the presence of atmospheric oxygen or pure O_2_ (1 atm) (Figure S7). On the other hand, oxygen saturation affects the efficiency of the subsequent steps of the reaction, suggesting that the conversion from Cu^I^ to Cu^II^ remains the rate-limiting step of the oxidation of catechols [22,27,28]. Furthermore, the addition of ascorbate during the first seconds of the reaction leads to the quenching of the absorption band at 300 nm (Figure S8).

To obtain more insight into the mechanism, the oxidation of the electron-poor substrate 4-chlorocatechol was investigated to assess whether the redox potential of the substrate influences the reaction rate and the lifetime of the intermediate. When 4-chlorocatechol oxidation is mediated by [Cu^II^–Aβ_4–16_], the chromophore at 300 nm is again generated (Figure S9), and its decay rate, which reflects the reduction rate of the complex, has a similar trend to that observed with the other catechols.

Taken together, these data confirm the reaction mechanism previously proposed for the oxidation of catechols by copper complexes with other neuronal peptides [22,27,28]:

1 [Cu^2+^–Aβ] + catechol ⇄ [Cu^2+^–Aβ–catechol]
2 [Cu^2+^–Aβ–catechol] → [Cu^+^–Aβ] + semiquinone^+^
3 [Cu^+^–Aβ] + catechol ⇄ [Cu^+^–Aβ–catechol]
4 [Cu^+^–Aβ–catechol] + O_2_ → [Cu–Aβ–catechol–O_2_]
5 [Cu–Aβ–catechol–O_2_] → [Cu^2+^–Aβ] + quinone
6 2 semiquinone^+^ → catechol + quinone

For both [Cu^II^–Aβ_1−x_] and [Cu^II^–Aβ_4−x_], the rate-determining step of the mechanism is reaction 4, where the reduced form of the ternary [Cu^I^–Aβ–catechol] species reacts with O_2_ to generate the dioxygen adduct indicated as [Cu–Aβ–catechol–O_2_]. Since the reactions were started by the addition of copper(II), the transient formation of the ternary [Cu^II^–Aβ–catechol] complex by reaction 1 is observed. However, with [Cu^II^–Aβ_1−x_], the fast disappearance of the copper(II)–catecholato band at 300 nm is covered by the band of the products formed in the subsequent reactions that absorb in the same spectral range, as explained above. On the other hand, in the reaction of [Cu^II^–Aβ_4−x_] complexes the slow reduction of copper(II) is displayed by the vanishing of the band at 300 nm. In this case, the following slow reaction of the [Cu^I^–Aβ–catechol] intermediate with O_2_ leads to very slow growth of the products absorbing at both 300 and 475 or 401 nm.

The reactivity of [Cu^II^–Aβ_4−x_] and [Cu^II^–Aβ_1−x_] complexes then differs in two main aspects. Firstly, the ATCUN site in Aβ_4−x_ stabilizes copper(II), making its reduction thermodynamically disfavored (reaction 2). However, this complex is not redox-inert, and catechol is able to reduce the copper(II) center slowly because coordination occurs with deprotonation to catecholate anion, which is electron-richer and allows electron transfer to occur through an inner sphere process. Secondly, once reduced to copper(I), which is the least reactive intermediate and rules the rate of the whole process through reaction 4, the reactivity of the two complexes is again different, with [Cu^I^–Aβ_4−x_] being less efficient to promote catechol oxidation. This difference is probably due to a different conformation of the Aβ_4−x_ peptide, which makes the coordination of copper(I) by histidines 13 and 14 less flexible and prone to leave access by catechol and molecular oxygen. Indeed, we showed previously [22] that the linear copper(I) coordination through His13 and His14 by Aβ_1−x_ must be distorted upon catechol coordination to allow a fast oxygenation reaction.

### 2.2. Catechol Oxidase Activity and Characterization of the Secondary Cu–His_2_ Binding Site of Aβ_4-16_

Besides the primary ATCUN binding site for copper(II), the histidines 13 and 14 can be involved in the metal interaction, under excess of copper(II), and this secondary site would be reducible to copper(I) by ascorbate [13,29]. Initially, the catechol oxidase reactivity of the dinuclear complex obtained in the presence of a second equivalent of copper ([Cu^II^_2_–Aβ_4–16_]) was assayed at low concentration of MC to detect reactivity changes attributable to the secondary Cu^II^ site. As shown before, in conditions of substrate subsaturation, the metal reduction rate is depressed, allowing better assessment of the oxidase reactivity of copper(II) bound to the low-affinity His-tandem binding site.

**Figure 5:**
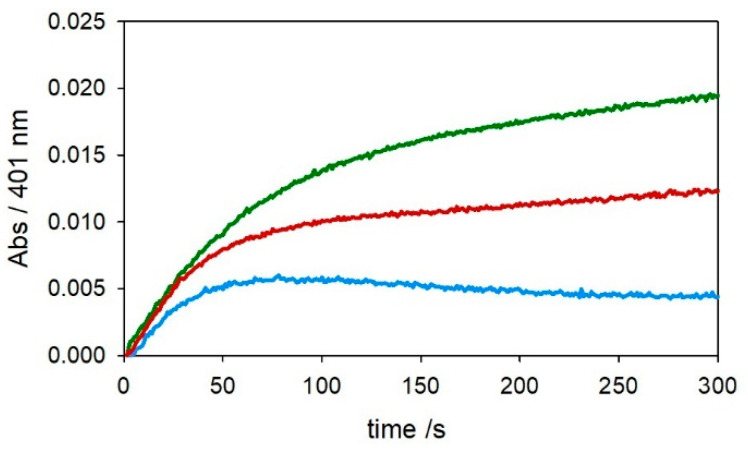
Kinetic profiles of MC (3 mM) oxidation with time in 50 mM HEPES buffer at pH 7.4 and 20 °C in the presence of Cu^II^ alone (25 μM, green trace). The same experiments were also performed upon the addition of [Cu–Aβ_4–16_] at 1:1 molar ratio (25 µM, light blue) and [Ni–Cu–Aβ_4–16_] at 1:1:1 molar ratio (25 µM, red).

As shown in Figure S10, the addition of 2 equiv. of copper(II) to Aβ_x−16_ peptides enhances the oxidation rate of catechol, but these data do not exclude the eventual participation of free copper(II) in solution acting as catalyst. Assuming a binding constant between copper(II) and the Aβ “secondary site” around 5 × 10^6^ M^−1^ at pH 7.4 [9], which matches with the value obtained for the interaction between copper(II) and catechol [30], a competitive interaction for the metal together with the low reactivity of the Cu–His_2_ site does not allow an active role of this site to be assessed in the reaction conditions. For these reasons, we chose to use another metal, nickel(II), to block the ATCUN site for a better characterization of the secondary site. Indeed, the copper(II) binding site in the ATCUN motif can easily accommodate nickel(II) ions with the same coordinative environment [29,31]. Therefore, in order to better isolate the reactivity of the Cu^II^–His_2_ site, 1 equiv. Ni^II^ was used to hinder the binding of the primary site to Cu^II^. As shown in Figure 5 (red trace), when [Ni^II^–Aβ_4–16_] is generated and 1 equiv. Cu^II^ is added, the competition for the metal between the peptide and the catechol at saturating concentration leads to a distribution between Cu^II^–catecholato complex and Cu^II^–His_2_ at the Aβ_4–16_ secondary site, resulting only in partial quenching of oxidase reactivity of the complex compared with free copper(II).

**Figure 6:**
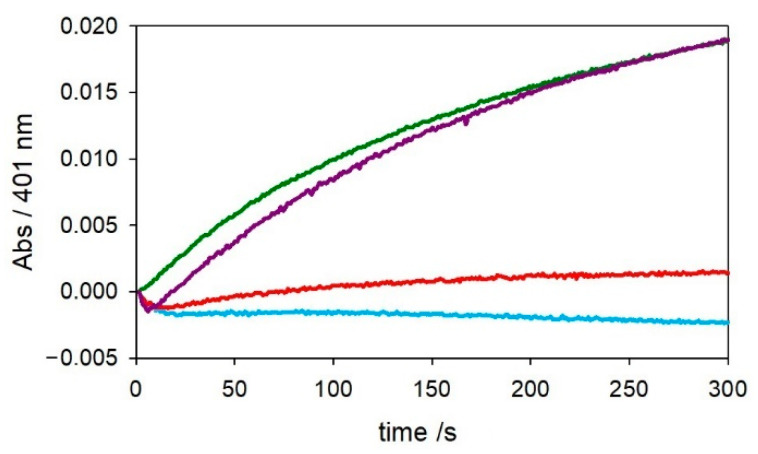
Kinetic profiles of MC (0.3 mM) oxidation with time in 50 mM HEPES buffer at pH 7.4 and 20 °C in the presence of Cu^II^ alone (25 μM, green trace). The same experiments were also performed upon the addition of Aβ_4–16_ complexes: [Cu–Aβ_4–16_] at 1:1 molar ratio (25 µM, light blue), [Ni–Cu–Aβ_4–16_] at 1:1:1 molar ratio (25 µM, red) and at 2:1:1 molar ratio (25 µM, violet).

On the other hand, when catechol oxidation is studied at low substrate concentration, the observed reactivity of [Ni^II^–Cu^II^–Aβ_4–16_] is negligible (Figure 6-red trace), suggesting two possible situations: If copper(II) replaces nickel(II) in the *N*-terminal site, nickel(II) ions will be free in solution but redox-inert and will not give rise to catechol oxidation. However, if nickel(II) is stably anchored to the ATCUN site, unbound copper(II) should be able to oxidize the substrate, and therefore, the observed redox quenching indicates the existence of an additional binding site for copper(II), in which the redox cycling is almost blocked. Moreover, the addition of a further equiv. of copper(II) restores the reactivity (violet trace in Figure 6), suggesting that the first equiv. of copper(II), after reduction to Cu^I^, is redox quenched in the His-tandem site while the second one in solution is free to catalyze the oxidative reaction.

**Figure 7:**
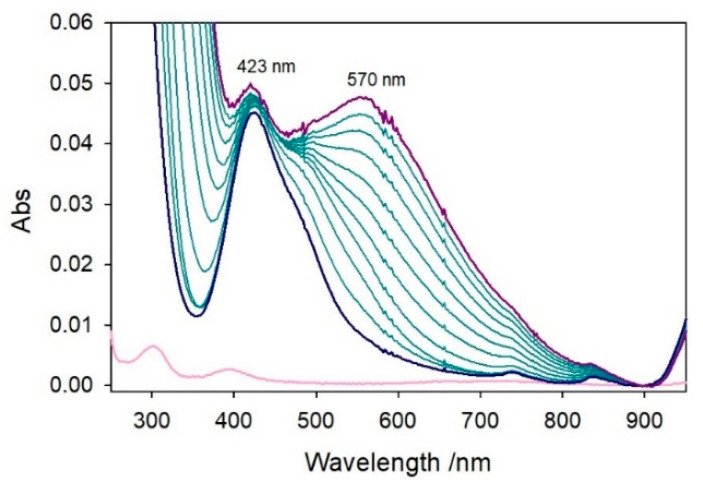
Titration of nickel(II)–Aβ_4–16_ complex (blue spectrum, at 1:1 ratio, 0.5 mM) with 0.0–0.55 mM copper(II) (final point as violet spectrum) in 5 mM phosphate buffer solution at pH 7.4. The pink spectrum corresponds to the absorption of free nickel(II).

To exclude the first hypothesis, the [Ni^II^–Aβ_4–16_] complex (0.5 mM) was titrated with copper(II) (0–0.55 mM) in 5 mM phosphate buffer solution at pH 7.4, and the corresponding optical spectra are shown in Figure 7. The spectra initially show a band at 423 nm due to the nickel(II)–peptide complex that is not influenced by addition of copper(II) and persists till the end of titration without significant changes. Upon addition of copper(II), another band at a longer wavelength (near 570 nm) develops with characteristics attributable neither to the typical Cu^II^–ATCUN complex, which shows a specific absorption at 525 nm [31,32], nor to free copper(II) in solution. Therefore, together with the previous kinetic data, when the *N*-terminal site is occupied by nickel(II), copper(II) binds to an additional site with spectroscopic properties [33] different from those of the Cu^II^–ATCUN site and in which its redox activity is almost silent. The stability of the 423-nm band also evidences that once Ni^II^ is inserted into the *N*-terminal site, no competition by copper(II) is exerted and Cu^II^ binding does not influence the coordination of Ni^II^, probing the existence of two independent and coordinatively different sites in the mixed metal complex.

When the titration is performed starting from [Cu^II^–Aβ_4–16_] and adding Ni^II^, the *N*-terminal Cu^II^–ATCUN complex with absorption at 525 nm [29] initially observed is not affected by nickel(II) ions, probing the stability of the Cu^II^ coordination set (Figure S11). UV-Vis spectra in Figure S12 testify to the stability of the *N*-terminal coordination of Ni^II^ (panel A) and Cu^II^ (panel B) in the ATCUN motif. However, while insertion of Cu^II^ into the ATCUN site is immediate, that of Ni^II^ is slower and, once the complex has been generated, is completely stable. In fact, both complexes are stable without changes in their optical spectra upon 24 h of incubation in the presence of the competitive metal. It is therefore clear that an equilibrium between the two species cannot be established and, upon adding further metal ions, these are unable to compete with or destabilize the initially bound ion.

### 2.3. CD and NMR Studies of the Binary and Ternary Complexes with Ni^II^ and Cu^II^ Bound to Aβ_4−x_ and Aβ_1−x_

**Figure 8:**
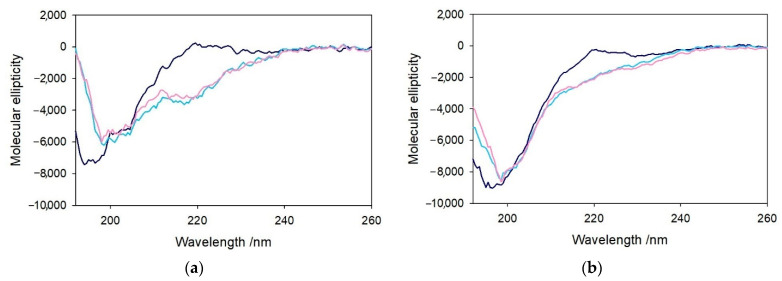
Far-UV CD spectra of 1.1 equiv. Aβ_4–16_ peptide (10 µM, panel **a**) and 1.1 equiv. Aβ_4–28_ peptide (5.5 µM, panel **b**) in 5 mM phosphate buffer solution at pH 7.4 (blue traces) and upon the addition of copper(II) (1 equiv., light blue) and DA (1 equiv., pink).

The previous observations were reinforced by the characterization of these complexes by circular dichroism, indicative of the conformation of the peptides in the far-UV region and the properties of the metal complexes in the visible region. The structural modifications of Aβ_4−x_ induced by the metal and eventually the substrate binding were assayed upon the addition of 1 equiv. of copper(II) to the peptide and the further addition of catechol (1 equiv.). The CD spectra shown in Figure 8 indicate an unstructured conformation of both Aβ_4–16_ and Aβ_4–28_ peptides in aqueous *medium*, as already observed for the corresponding Aβ_1−x_ homologs [25].

The addition of a stoichiometric amount of copper(II) induces some local changes of the peptides (light blue spectrum), while the addition of catechol (pink spectrum) does not significantly affect the conformation of the peptide backbone. Control experiments on the related Aβ_1−x_ peptides were also performed to verify the absence of relevant structural changes upon addition of copper(II), and, in this case, the formation of the metal complexes only results in a modest decrease in the chain mobility (Figure S13).

**Figure 9:**
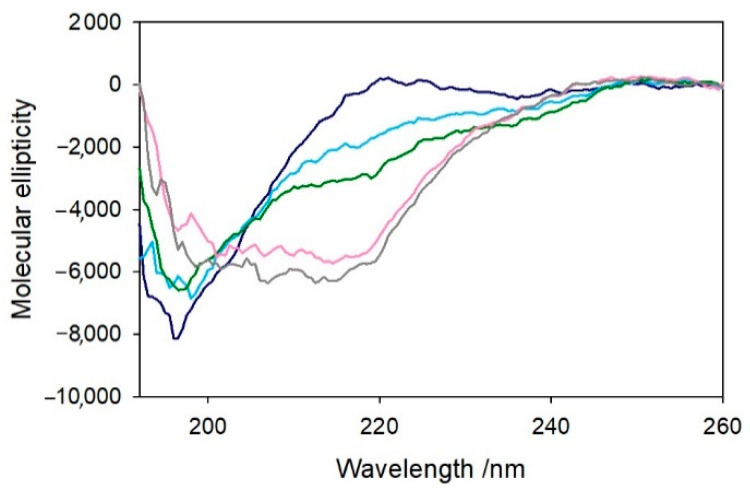
Far-UV CD spectra of Aβ_4–16_ peptide (10 µM) in 5 mM phosphate buffer solution at pH 7.4 (blue trace) and upon the addition of Ni^II^ (9.5 µM), after few seconds of incubation (light blue) and 15 min (green). Then, 1 (pink) and 2 equiv. of copper(II) (grey) were added to the Ni–Aβ_4–16_ complex.

A somewhat more significant conformational rearrangement was observed when 1 equiv. Ni^II^ was added to Aβ_4–16_ in the same buffer solution (Figure 9). An initial spectrum was recorded upon addition of the metal ion to the peptide solution (light blue spectrum), and a second recording was made after 15 min of incubation (green spectrum). This behavior suggests a gradual structuring of the peptide dependent on metal binding which was not observed in the case of copper(II). When Cu^II^ is added to Aβ_4–16_, a (modest) conformational change immediately occurs without any relevant variation with time (Figure S14), while in the case of Ni^II^, the partial folding of the peptide shows a slower kinetics and leads to a slightly different CD spectral shape. Moreover, the addition of 1 equiv. Cu^II^ to [Ni^II^–Aβ_4–16_] (Figure 9-pink spectrum) significantly alters the peptide conformation, enhancing the intensity of the band at 220 nm and decreasing the peak at 198 nm. The resulting spectrum is different from that of the Cu^II^–Aβ_4–16_ complex, showing that the simultaneous binding of the two metals occurs without displacement of Ni^II^ from the primary binding site, while binding of Cu^II^ occurs in a secondary site. The further addition of a second equivalent of Cu^II^ (Figure 9-grey spectrum) shows negligible effects in the CD spectrum, indicating that it cannot bind to the peptide and, therefore, remains free in solution.

**Figure 10:**
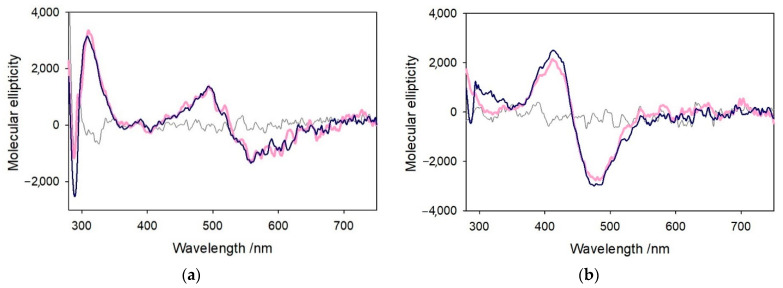
(**a**) Vis-CD spectra of of copper(II) (2 mM) alone in 5 mM phosphate buffer solution at pH 7.4 (grey trace) and upon the addition of 1 equiv. Aβ_4–16_ peptide (2 mM, pink) and further addition of 1 equiv. of Ni^II^ to the initial complex (blue). (**b**) Vis-CD spectra of nickel(II) (2 mM) alone (grey trace) and upon the addition of 1 equiv. Aβ_4–16_ peptide (2 mM, pink) and further addition of 1 equiv. of Cu^II^ (blue).

The coordination of Cu^II^ and Ni^II^ to Aβ_4–16_ was also characterized by CD in the near-UV and visible regions, as shown in Figure 10. The spectrum of [Cu^II^–Aβ_4–16_] was recorded in phosphate buffer solution at pH 7.4 (pink spectrum) after 30 min of incubation. The same procedure was followed in the reverse order, preforming [Ni^II^–Aβ_4–16_] and then adding Cu^II^. The CD spectrum of [Cu^II^–Aβ_4–16_] shows a positive peak at 310 nm that is attributable to amide–Cu^II^ LMCT [24], while the d-d bands around 490 and 560 nm suggest the presence of 3N or 4N coordination in a square planar structure of the metal [34], matching that of the Cu^II^–ATCUN site. On the other hand, the CD peaks at 412 and 478 nm shown by [Ni^II^–Aβ_4–16_] are typical of 4N {NH_2_, 2N^−^, N_Im_} equatorial coordination in a similar square planar geometry [35]. The CD spectra of both complexes remain unchanged when a second metal ion, Ni^II^ and Cu^II^, respectively, is added. Thus, the metal ion initially bound to the *N*-terminal fragment of the peptide is not displaced by the other metal ion subsequently added, indicating the lack of competition for the primary site. It is interesting to note that binding of copper(II) to the secondary site of [Ni^II^–Aβ_4–16_] occurs without appreciable CD changes, indicating the absence of CD contribution by chelate effect, due to the fact that copper(II) is bound linearly to the two histidines and with water bound in the remaining equatorial positions. This arrangement allows rotation mobility of the imidazole rings around the N–Cu–N axis averaging out the contribution to CD by vicinal effects.

To probe the involvement of the His-tandem residues in the binding of copper(II) when the primary site is not available, a solution of Aβ_4–16_ in deuterated phosphate buffer solution at pH 7.4 was prepared (8.6 mM, 1.1 equiv.), and the ^1^H-NMR spectrum of peptide alone was acquired as control. Then, Ni^II^ (7.8 mM, 1 equiv.) was added to block the ATCUN site, and after 30 min, Cu^II^ (78 µM) was added to the previous complex. This substoichiometric amount of copper(II) guarantees the observation of the effect of the interaction with the peptide without an excessive broadening of the signals. To assign each signal to the protons of amino acid residues of the amyloid fragment, ^1^H-COSY spectrum of Aβ_4–16_ with the same concentration was recorded (Chart S1 and Figure S15). The spectral data suggest that Ni^II^ and Cu^II^ strongly affect the imidazole proton signals of all three histidines, indicating the involvement of His6 in Ni^II^ binding and His13 and His14 in the interaction with Cu^II^ (Figure S16). No detectable changes occur for the signals attributable to Phe4 and Tyr10 residues. In the aliphatic region shown in Figure S17, the more perturbed signals upon addition of the metal ions are those of γ-protons of Gln15 at 2.14 ppm, which show an evident paramagnetic effect, together with a slight broadening of the signals corresponding to Hα, Hγ, and Hδ of Val12 at ~3.75, 0.72, and 0.60 ppm. The Ni^II^ and Cu^II^ ions do not affect signals of either Ser8 or Tyr10, as shown by the two triplets given by their alpha protons around 4.3 ppm. The overlaid signals of α-protons at 4.12 ppm of Arg5 and Lys16 residues are both affected by the metal ions, while β-protons of Asp7 are shifted after the addition of nickel(II) but also seem to be influenced by the presence of copper(II). In conclusion, the data support the localization of copper(II) in the *C*-terminal region encompassing residues 12 to 16, when Ni^II^ is placed in the *N*-terminus, showing modest effects on Arg4, His6, and Asp7. Moreover, the spectrum of this dinuclear [Ni–Cu–Aβ_4–16_] complex is stable with time, as shown by ^1^H-NMR spectra recorded after 6 and 12 h of incubation (Figure S18). Thus, no exchange between the two metals occurs in these conditions.

## 3. Discussion

The redox reactivity of copper(II) bound to amyloid-β fragments Aβ_1−x_ and Aβ_4−x_ was studied in terms of their oxidative reactivity toward catecholamines with a dual purpose: (i) to verify the currently assumed redox-inertness of the metal ion when trapped in the ATCUN site and (ii) to investigate the role of the two vicinal histidines, His13 and His14, when the peptide binds a second copper(II) ion. It has been suggested that Aβ truncated fragments with Phe4 at the *N*-terminal act as strong ligands for copper(II) ions, resulting in highly stable complexes in which the metal ion is totally unreactive [13,15]. This assumption is contradicted by the evidence shown in this paper; i.e., redox changes of Cu^II^–Aβ_4−x_ can be induced through the binding of dopamine, or other catechols, leading to a modest, but not negligible, oxidase activity depending on the concentration of the substrates. Other recent studies investigated the contribution of a secondary coordination site for copper(II) at the His-tandem residues and the possible contribution to oxidative reactivity in the presence of copper(I) [14,16].

As we previously reported [22,27,28], the general mechanism of catecholamine oxidation promoted by Cu^II^–Aβ complexes follows a biphasic catalysis, in which the initial reduction of the metal center by the catechol to give the Cu^I^–Aβ species is followed by reoxidation by dioxygen in the rate-determining step of the process. When copper(II) is anchored to the ATCUN site, the stabilization of the Cu^II^ state decreases the rate of the first reaction, limiting the rate of redox cycling of Cu^II^–Aβ_4−x_ complexes. As shown here, the binding of catechol as an external ligand to the axial position of the square planar Cu^II^–ATCUN complex allows the gradual reduction to Cu^I^, accompanied by the vanishing of the optical signature at 300 nm. In addition, the reduced form, which rules the rate of the process through the slow oxygenation reaction, is less reactive if compared to the species generated by Aβ_1−x_ peptides. On the contrary, as shown in our previous studies on Cu–Aβ_1–x_ complexes [22,25,27], the Cu^II^ form is less stable in response to reduction; redox cycling is thus faster, and the catechol oxidation is more efficient. This results in faster production of oligomeric species with intense absorptions in the near-UV region, which overlap with the band at 300 nm, masking the sequential redox reactions.

Besides the levels of catecholamines, the amount of copper in the synaptic cleft is highly modulated in relation to the neurotransmission of signals, where the basal level around 3 µM can be rapidly enhanced to 100–250 µM upon the excitatory pulse [36]. Considering a micromolar to nanomolar concentration range for Aβ molecules [36], it is reasonable to assume a simultaneous interaction of Aβ peptides with multiple copper ions in concomitance with the pulse. Besides the binding site for Cu^II^ provided by the ATCUN motif, Aβ truncated forms contain the two vicinal His13 and His14 residues that are considered as binding sites of Cu^I^ ions [29]; thus, these peptides can easily accommodate the metal ion in both redox states. The redox activity of Cu^II^ bound to the His-tandem site has been recently suggested in which copper(II) can be quickly reduced to copper(I) via reaction with ascorbate [29], but the characterization of the redox process is still incomplete. To investigate the oxidative reactivity of copper(II) bound to this secondary site of the peptides, we chose to occupy the ATCUN site with an additional metal, namely nickel(II) ion, which is redox-inert and can be bound strongly by the *N*-terminal ATCUN site [31]. Other metal cations, also detected as circulating free ions in the synaptic cleft, such as zinc(II) ions [37], were discarded due to their lower stabilization by the ATCUN site, in agreement with the different coordinative preference of this ion, which is typically found in sites of tetrahedral geometry, as in carbonic anhydrase [38].

When Ni^II^ is coordinated in the *N*-terminal domain of Aβ truncated peptides, additionally added copper(II) ion shifts to the His_2_ site, and no exchange of metal between the two binding sites is established. This mixed metal [Ni^II^–Cu^II^–Aβ_4−x_] complex exhibits no catechol oxidase activity, probably due to the restricted mobility of the peptide chain around the Cu–His_2_ site when the *N*-terminal site is immobilized by nickel(II) ion. In principle, it could be assumed that this secondary His-tandem site could be used for scavenging of copper ions “chelatable” in solution, preventing their redox reactivity.

In conclusion, the protective role of truncated amyloid-β peptides against Cu^II^/Cu^I^ redox changes that was previously assumed turns out to be reduced by the results of our investigation. Catecholamines are in fact good ligands for copper and redox-active molecules that can reduce copper(II) even when strongly stabilized by the ATCUN site, provided that the metal can be accessed through an available coordination position. However, it should be added that much more than for [Cu^II^–Aβ_1−x_] complexes, the redox reactivity of [Cu^II^–Aβ_4−x_] complexes becomes appreciable when the concentration of the catecholamine reaches significant levels. On the other hand, when the copper(II) ion is confined in the secondary His-tandem site of truncated Aβ_4−x_ peptides, its redox reactivity is completely quenched. These data provide additional information about the chemistry of Cu^II^–Aβ complexes, highlighting the potential role of these fragments in the homeostatic control of copper in the brain. The full comprehension of the molecular events on the onset of this pathology can direct future research towards the development of new drugs for AD treatment [39,40].

## 4. Materials and Methods

Protected amino acids, rink amide resin, and other reagents for peptide synthesis, were purchased from Novabiochem (Milan, Italy); all other reagents were from Sigma Aldrich (St. Louis, MO, USA).

### 4.1. Peptide Synthesis

Aβ_1–16_ (_1_DAEFRHDSGYEVHHQK_16_-NH_2_), Aβ_4–16_ (_4_FRHDSGYEVHHQK_16_-NH_2_), Aβ_1–28_ (_1_DAEFRHDSGYEVHHQKLVFFAEDVGSNK_28_-NH_2_), and Aβ_4–28_ (_4_FRHDSGYEVHHQKLVFFAEDVGSNK_28_-NH_2_) peptides were synthesized using the traditional fluorenyl methoxycarbonyl (Fmoc) solid-phase synthesis in DMF [41]. The growth of peptide sequence occurred on the rink-amide resin MBHA (substitution 0.78 mmol/g) as polymeric support, so as to have an amidated *C*-terminal product. The cleavage from the resin was performed through a solution of TFA (95%), TIS (2.5%), and water (2.5%), and the precipitation of the product was achieved by addition of cold diethyl ether. The resulting powder was then purified through HPLC and was eluted using a 0−100% linear gradient of 0.1% TFA in water to 0.1% TFA in CH_3_CN over 50 min (flow rate of 4 mL/min, loop 2 mL), as eluent. The product was lyophilized and controlled by direct injection in mass spectrometry, obtaining the following ESI-MS data: Aβ_1–16_ *m*/*z* 1955(+), 978(2+), 652(3+), 489(4+); Aβ_4–16_ *m*/*z* 1641(+), 821(2+), 547.7(3+), 411(4+); Aβ_1–28_ *m*/*z* 1631(2+), 1088(3+), 816(4+), 653(5+); Aβ_4–28_ *m*/*z* 1474(2+), 983(3+), 737.5(4+), 590.2(5+).

### 4.2. Catalytic Oxidation of Catechol Substrates

The oxidation of catechols catalyzed by copper complexes was studied at 20 °C in 50 mM HEPES buffer at pH 7.4 with an Agilent (Santa Clara, CA, United States) 8453 diode array spectrophotometer, equipped with a thermostated, magnetically stirred optical cell. The reactions of copper(II) nitrate (25) alone or bound to the amyloid-β fragments (25–50 µM) was assayed by UV-Vis spectroscopy for 300 s following the dopaminochrome generated from dopamine at 475 nm, the 4-methylquinone derived from 4-methylcatechol at 401 nm, or the 4-chloroquinone derived from 4-chlorocatechol at 450 nm. The substrate concentration was varied from subsaturating (0.3 mM) to saturating values (3 mM). The substrate autoxidation was also taken into account and subtracted from the spectrophotometric traces. To assay the presence in solution of oligomeric species of Aβ_4–28_, 2 mg of peptide was dissolved in 1 mL hexafluoroisopropanol (HFIP) and stirred for at least 3 h to dissolve the small aggregates; the solution was then lyophilized and the peptide was immediately used [25]. When the redox behavior of bis-His tandem site was assayed, 2 equivalents of copper(II) (50 µM) were used or a copper(II):nickel(II) (1:1, 25 µM) mixture was preincubated with the peptide solution to preform the dinuclear complex [Ni^II^–Cu^II^–Aβ_4–16_], and then the reactivity of the complex was investigated. Finally, the influence of oxygenation rate on the efficiency of copper redox cycling was examined through the presaturation of buffer solution with pure dioxygen (1 atm) compared to solution exposed to the air. The quantification of peptide stock solutions was obtained by the absorbance of the solutions at 280 nm corresponding to the tyrosine band (ε 1480 M^−1^ cm^−1^) [42]. All measurements were performed at least in duplicate.

### 4.3. UV-Visible Characterization of Dinuclear Metal–Peptide Complexes

The titration of [Ni^II^–Aβ_4–16_] (1:1, 0.5 mM) in the presence of copper(II) was performed at 20 °C in phosphate buffer solution at pH 7.4 in 1 cm path length cell. Copper(II) nitrate was added in the concentration range 0–0.55 mM. The same conditions were maintained for the titration starting from [Cu^II^–Aβ_4–16_] and adding Ni^II^ up to 1.1 equivalent.

### 4.4. Oxidative Modification of N-Terminally Truncated Aβ Peptides via HPLC-MS

The competitive modification of Aβ_4–28_ during catechol oxidation was analyzed by Thermo-Finnigan (San Jose, CA, United States) HPLC-ESI/MS using an LCQ ADV MAX ion-trap mass spectrometer. The solutions from the previous kinetic experiments were fractionated into aliquots and cooled in liquid N_2_ at specific reaction times (15, 25, 35, 60 min). Therefore, the reaction mixtures containing copper(II) nitrate (25 µM), Aβ_4–28_ (25 µM), and MC or DA (3 mM) in 50 mM HEPES buffer at pH 7.4 were eluted by using 0.1% HCOOH in distilled water (solvent A) and 0.1% HCOOH in acetonitrile (solvent B), with a flow rate of 0.2 mL/min. Elution started with 98% solvent A for 5 min followed by a linear gradient from 98 to 55% A in 65 min.

### 4.5. Characterization via Circular Dichroism and Nuclear Magnetic Resonance of Metal–Aβ Complexes

The secondary structure of Aβ_4−x_ and Aβ_1−x_ peptides was studied by CD in the range from 192 to 260 nm using a Jasco (JASCO International Co. Ltd., Hachioji, Tokyo, Japan) 1500 spectropolarimeter in 1 cm path length cell. Micromolar solutions of peptides were studied in 5 mM phosphate buffer at pH 7.4 and seven acquisitions were performed. Copper(II) nitrate and nickel(II) nitrate were added in a molar ratio of 1:1 with respect to the peptide. To allow homogenization and equilibration of the solution, each spectral recording was carried out after 30 min from the addition of the metal solution. The visible spectra were obtained in the same conditions, but using higher concentrations of the complexes (2 mM) and following the spectral region encompassing 280–750 nm. The NMR experiment was performed by dissolving Aβ_4–16_ in D_2_O and recording an initial spectrum of the peptide (8.6 mM) in 5 mM deuterated phosphate buffer solution at pH 7.4. Ni^II^ (7.8 mM) was then added to the NMR tube and the acquisition was obtained after 30 min of incubation. A small amount of Cu^II^ (78 µM) was finally added to avoid the loss of resolution of the entire spectrum. The acquisition was repeated after 6 and 12 h.

## Supplementary Materials

UV-visible spectra and kinetic profiles, HPLC-MS chromatograms and related tables, and CD and NMR spectra are available online at https://www.mdpi.com/article/10.3390/ijms22105190/s1.
