# Supplementary material for: Oxidase Reactivity of CuII Bound to N-Truncated Aβ Peptides Promoted by Dopamine
Source: Int J Mol Sci. 2021 May 14;22(10):5190. doi: 10.3390/ijms22105190 (PMC8155989; doi:10.3390/ijms22105190)
Supplement: Supplementary file 1 [file ijms-22-05190-s001.zip › sup.pdf]

Supporting Information

Oxidase reactivity of Cu^{II} bound to *N*-truncated Aβ peptides promoted by dopamine

Chiara Bacchella, Simone Dell'Acqua, Stefania Nicolis, Enrico Monzani, and Luigi Casella*

Dipartimento di Chimica, Università di Pavia, Via Taramelli 12, 27100 Pavia, Italy
* Correspondence: luigi.casella@unipv.it.

General. All compounds were purchased from Sigma-Aldrich. Preparative HPLC used for the peptide purification was carried out with a Shimadzu LC-20AD Prominence instrument equipped with a diode array detector. Mass spectra and LCMS/ MS data were obtained with a LCQ ADV MAX ion-trap mass spectrometer equipped with an ESI ion source. The system was run in automated LC-MS/MS mode and using a surveyor HPLC system (Thermo Finnigan, San Jose, CA, USA) equipped with a Phenomenex Jupiter 4u Proteo column (4 µm, 150×2.0 mm). For the study of oxidative modifications, Bioworks 3.1 and Xcalibur 2.0.7 SP1 software were used (Thermo Finnigan, San Jose, CA, USA). UV-Vis spectra and kinetic experiments were recorded on an Agilent 8453 diode array spectrophotometer, using an optical cell with 1 cm path length. NMR spectra were recorded on a Bruker AVANCE 400 spectrometer. Circular dichroism (CD) spectra were recorded with quartz cell of 1 cm path length for UV and Vis regions, using a Jasco J-1500 spectropolarimeter.

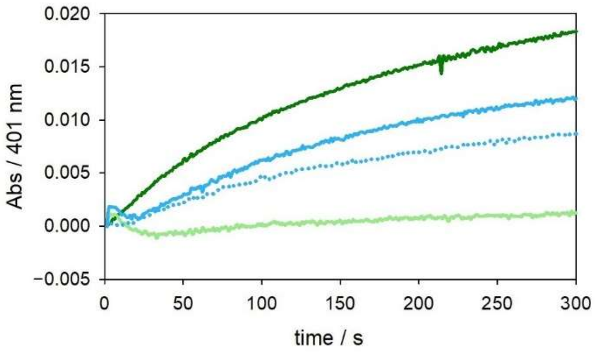

Figure S1. Kinetic profiles of MC (0.3 mM) oxidation with time in 50 mM HEPES buffer at pH 7.4 and 20 °C in the presence of Cu^{II} alone (25 µM, green trace) and with 1 equiv. $A\beta_{4-28}$ dissolved in water (25 µM, solid light blue), 1 equiv. (25 µM, dotted light blue) and 1.2 equiv. $A\beta_{4-28}$ (30 µM, solid light green) treated with HFIP for 3 hours before the preparation of the stock solution.

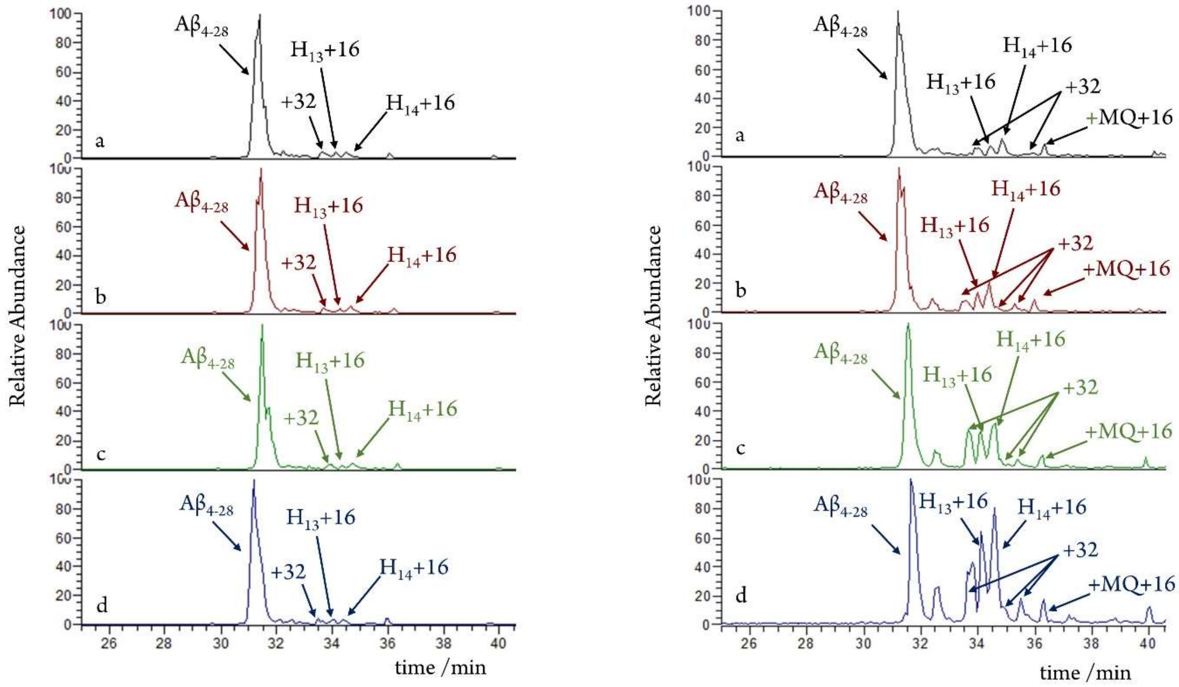

Figure S2. HPLC-MS elution profiles of $A\beta_{4-28}$ peptide (25 μM) in 50 mM HEPES buffer at pH 7.4 with DA (3 mM, left panel) or MC (3 mM, right panel) in the presence of Cu^{II} (25 μM). The reaction was monitored at different reaction times: 15 min (panel a), 25 min (panel b), 35 min (panel c) and 60 min (panel d). The insertion of *O*-atoms is indicated as +16, + 32 and +48 mass increments while +MQ corresponds to the covalent bound of quinone species generated during 4-methylcatechol oxidation to histidines.

Table S1. Modification with time of $A\beta_{4-28}$ peptide (25 μM) detected by LC/MS analysis upon reaction with MC (3 mM) in the presence of Cu^{II} (25 μM), in 50 mM HEPES buffer pH 7.4 at 20 °C.

	**$A\beta_{4-28}$ (unmodified)**	**$A\beta_{4-28}$ mono-oxidized** (+16)	**$A\beta_{4-28}$ bis-oxidized** (+32)	**$A\beta_{4-28}$ tris-oxidized** (+48)	**$A\beta_{4-28}$ -dopaminated** (+120, +122)	**$A\beta_{4-28}$ -oxidized and dopaminated** (+136, +138)
**Time**						
**15'**	66%	15%	4%	1%	6%	8%
**25'**	56%	20%	7%	1%	6%	10%
**35'**	49%	23%	8%	1%	6%	13%
**60′**	41%	29%	11%	1%	4%	14%

Table S2. Modification with time of A$\beta_{4\text{-}28}$ peptide (25 μM) detected by LC/MS analysis upon reaction with DA (3 mM) in the presence of Cu^{II} (25 μM), in 50 mM HEPES buffer pH 7.4 at 20 °C.

	A$\beta_{4\text{-}28}$ (unmodified)	A$\beta_{4\text{-}28}$ mono-oxidized (+16)	A$\beta_{4\text{-}28}$ bis-oxidized (+32)	A$\beta_{4\text{-}28}$ tris-oxidized (+48)	A$\beta_{4\text{-}28}$ -dopaminated (+145, +147, +149, +151)	A$\beta_{4\text{-}28}$ -fragmented
**time**						
**15'**	78%	14%	4%	1%	3%	-
**25'**	75%	17%	4%	1%	3%	-
**35'**	68%	20%	6%	1%	5%	-
**60′**	62%	17%	6%	2%	9%	4%*

*Main fragmentation sites located between E_{11}/V_{12} (1%); H_{13}/H_{14} (1%); D_7/S_8 (1%) and Q_{15}/K_{16} (1%)

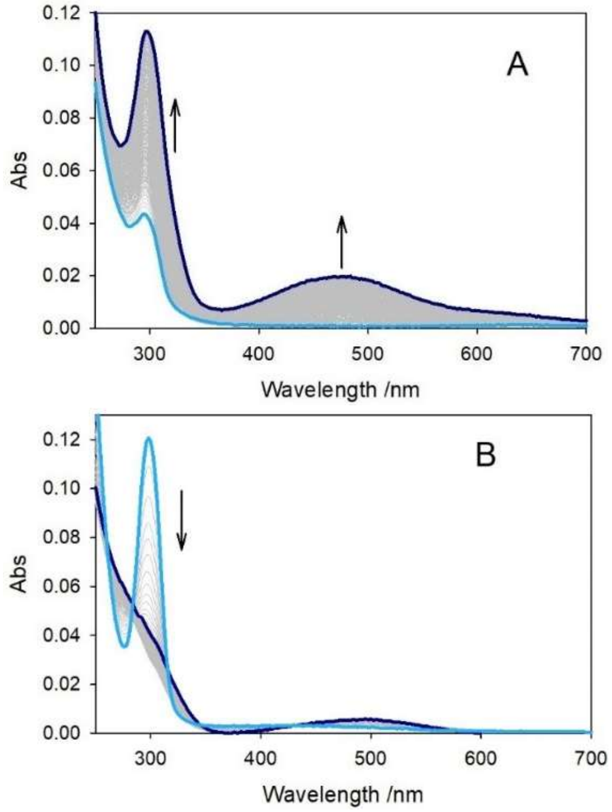

Figure S3. Absorbance profiles corresponding to the oxidation with time (starting point in light blue and final profile in blue) of DA (0.3 mM) catalyzed by [Cu-A$\beta_{1\text{-}16}$] (25 μM, panel A) and [Cu-A$\beta_{4\text{-}16}$] (25 μM, panel B). Spectra taken each 1 s for 300 s reaction time.

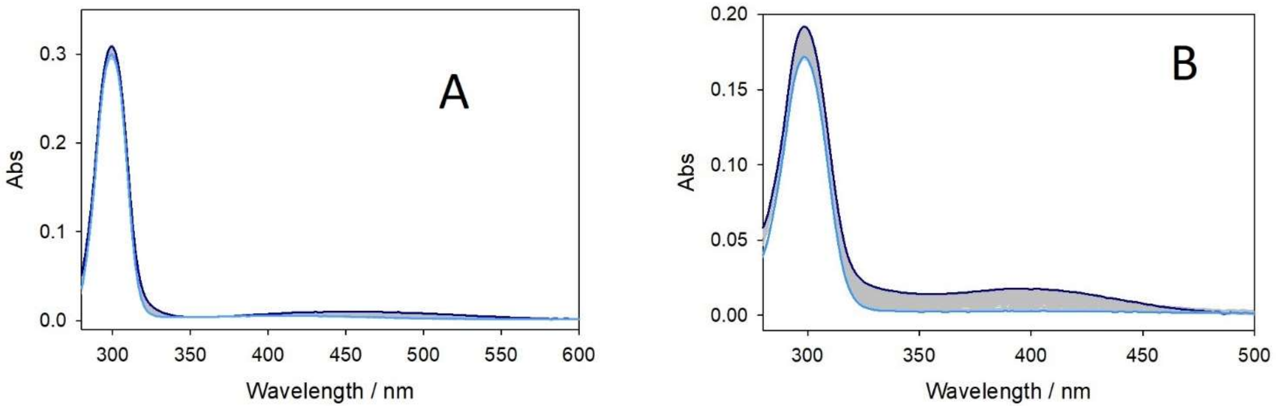

Figure S4. Absorbance profiles corresponding to the oxidation with time (starting point in light blue and final profile in blue) of DA (0.3 mM, panel A) and MC (0.3 mM, panel B) catalyzed by copper(II) alone (25 μM). Spectra taken each 1 s for 300 s reaction time.

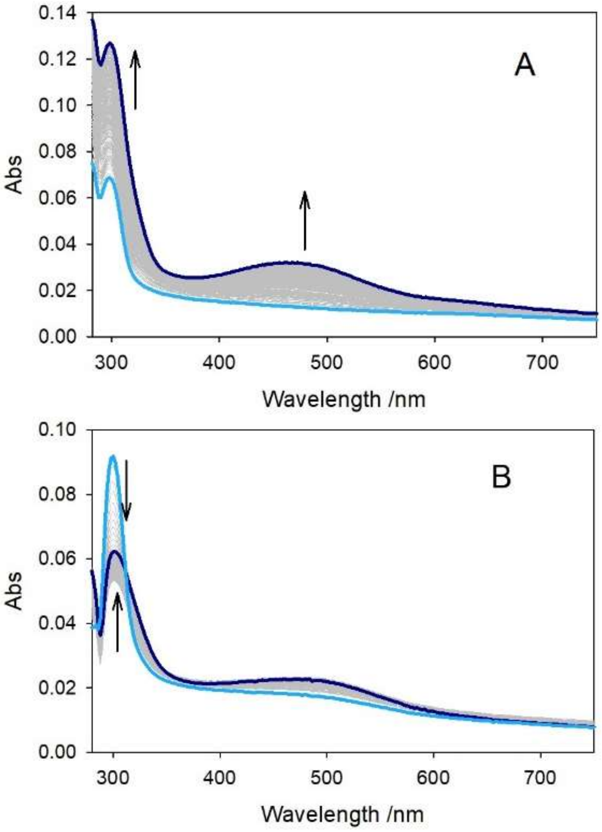

Figure S5. Absorbance profiles corresponding to the oxidation with time (starting point in light blue and final profile in blue) of DA (0.3 mM) catalyzed by [Cu-Aβ_{1-28}] (25 μM, panel A) and [Cu-Aβ_{4-28}] (25 μM, panel B). Spectra taken each 1 s for 300 s reaction time.

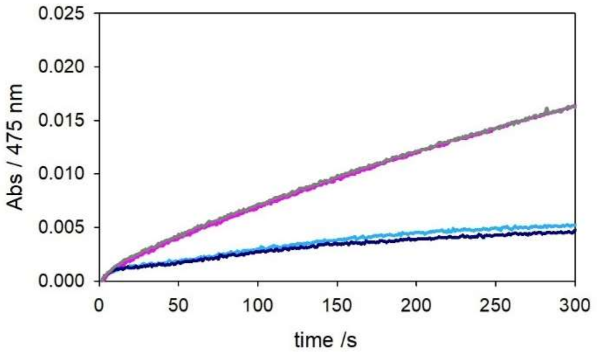

Figure S6. Kinetic profiles of DA (0.3 mM) oxidation with time in 50 mM HEPES buffer solution at pH 7.4 and 20 ° C in the presence of the following complexes at 1:1 molar ratio (25 μM) generated by the direct addition in the reaction *medium*, [Cu-A$\beta_{4\text{-}16}$] (light blue trace) and [Cu-A$\beta_{1\text{-}16}$] (pink), or upon the pre-incubation of peptide with copper, [Cu-A$\beta_{4\text{-}16}$] (blue) and [Cu-A$\beta_{1\text{-}16}$] (grey).

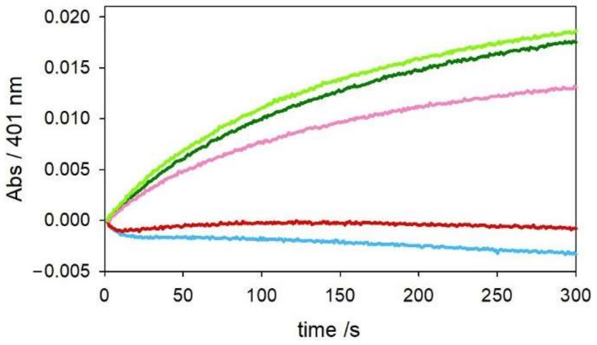

Figure S.7 Kinetic profiles of MC (0.3 mM) oxidation with time in 50 mM HEPES buffer at pH 7.4 and 20 °C in the presence of Cu^{II} alone (25 μM, green trace) and with 1 equiv. A$\beta_{1\text{-}16}$ (25 μM, pink) and 1 equiv. A$\beta_{4\text{-}16}$ (25 μM, light blue). Additional traces were obtained upon dioxygen saturation of the buffer solution and following the substrate oxidation catalyzed both by [Cu-A$\beta_{1\text{-}16}$] (light green) and by [Cu-A$\beta_{4\text{-}16}$] (red).

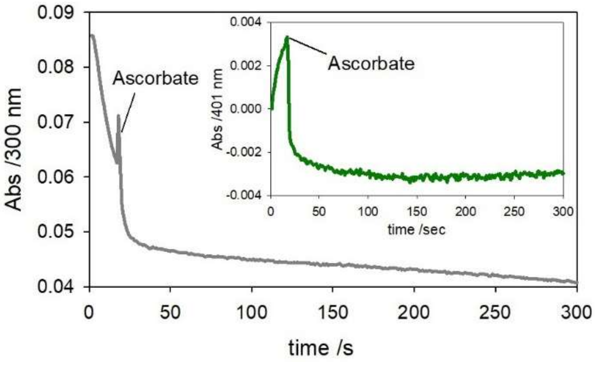

Figure S8. Kinetic profiles obtained at 300 nm (grey trace) and 401 nm (green) of 4-methylcatechol oxidation (3 mM) in 50 mM HEPES buffer at pH 7.4 catalyzed by [Cu-A$\beta_{4\text{-}16}$] (25 μM) and followed before and after the addition of ascorbate (50 μM).

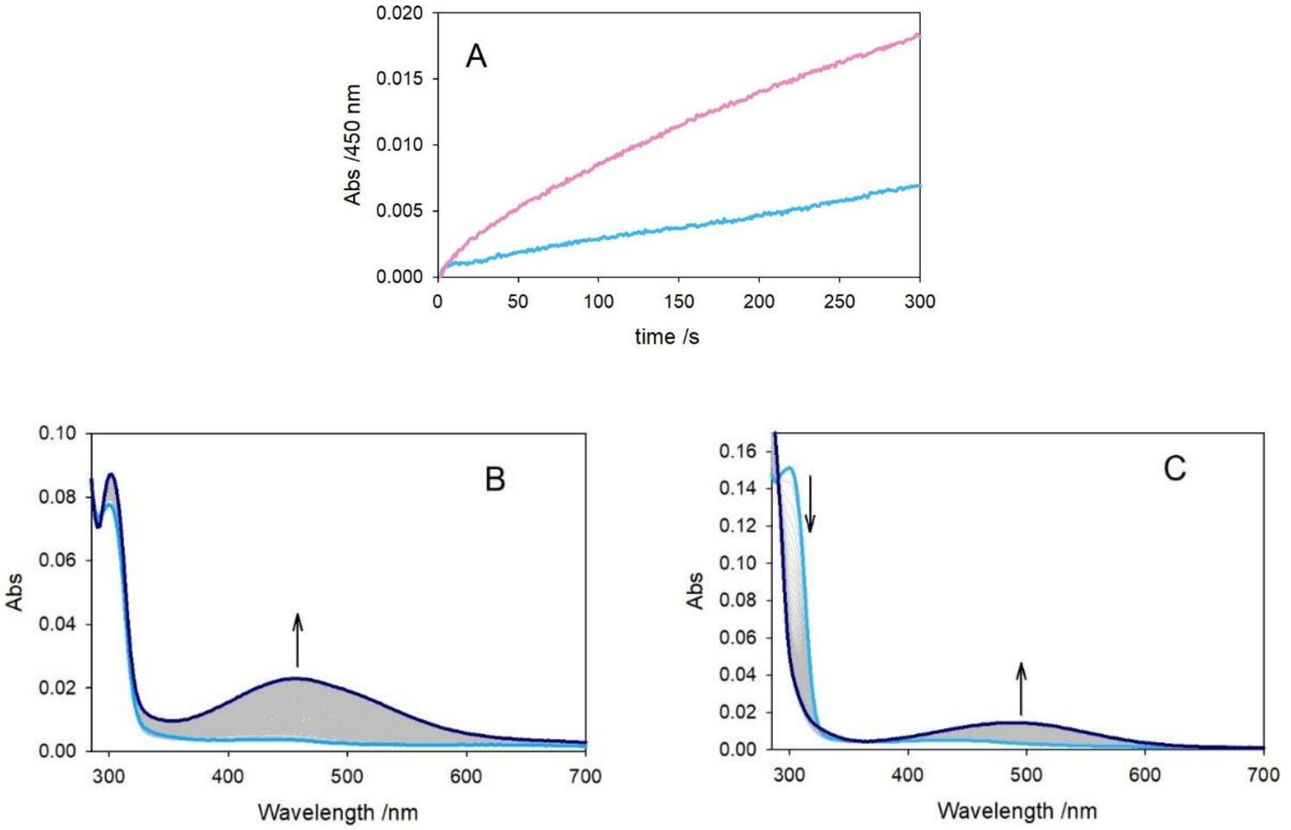

Figure S9. Kinetic profiles of 4-chlorocatechol (0.3 mM, panel A) oxidation with time in 50 mM HEPES buffer at pH 7.4 and 20 °C in the presence of [Cu-$A\beta_{1-16}$] (25 μM, pink trace) and [Cu-$A\beta_{4-16}$] (25 μM, light blue). Absorbance profiles corresponding to the oxidation with time (starting point in light blue and final profile in blue) of 4-cholocatechol are shown in the panel B for [Cu-$A\beta_{1-16}$] and panel C for [Cu-$A\beta_{4-16}$]. Spectra taken each 1 s for 300 s reaction time.

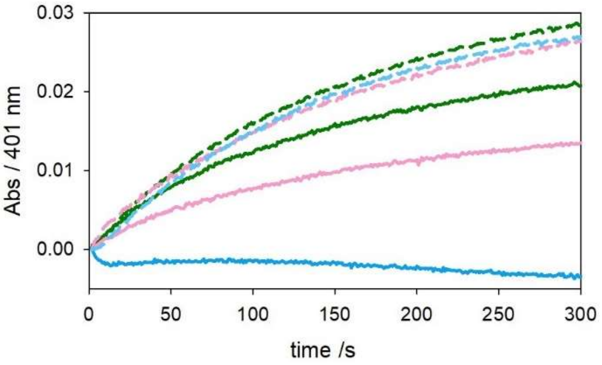

Figure S10. Kinetic profiles of MC (0.3 mM) oxidation with time in 50 mM HEPES buffer solution at pH 7.4 and 20 °C in the presence of only Cu^{II} (25 μM, solid green trace or 50 μM, dashed green) and with the following complexes: [Cu-$A\beta_{1-16}$] at 1:1 molar ratio (25 μM, solid pink), [Cu-$A\beta_{1-16}$] at 2:1 molar ratio (dashed pink), [Cu-$A\beta_{4-16}$] at 1:1 molar ratio (solid light blue) and [Cu-$A\beta_{4-16}$] at 2:1 molar ratio (dashed light blue).

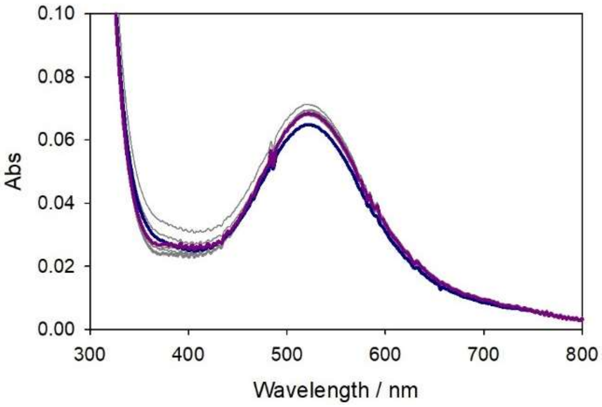

Figure S11. Titration of Cu-A$\beta_{4\text{-}16}$ complex (blue spectrum, 1:1 ratio, 0.5 mM) by the addition of Ni^{II} (0-0.5 mM, final point as violet spectrum) in 5 mM phosphate buffer solution at pH 7.4.

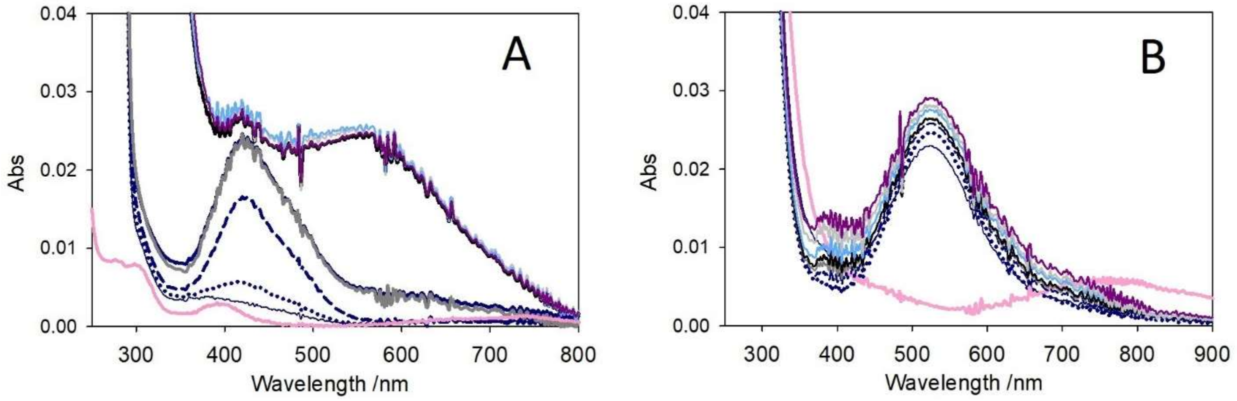

Figure S12. UV-vis characterization with time of: panel A) Ni^{II}-A$\beta_{4\text{-}16}$ complex at 1:1 ratio (0.24 mM) (at starting point, thin blue spectrum; upon 1 min of incubation, dotted blue; 5 min, dashed blue; 30 min, solid blue; 60 min, dark grey); 1 equiv. Cu^{II} was then added to the adduct (black) and the acquisition was repeated after the incubation of 30 min (light blue), 3h (light grey) and 24h (violet). The pink spectrum corresponds to the absorption of Ni^{II} alone. Panel B) Cu^{II}-A$\beta_{4\text{-}16}$ complex at 1:1 ratio (0.20 mM) (at starting point, thin blue spectrum; upon 5 min of incubation, dotted blue; 30 min, dashed blue; 60 min, dark grey); 1 equiv. Ni^{II} was then added to the adduct (black) and the acquisition was repeated after the incubation of 30 min (light blue), 30 min (light grey) and 24 h (violet). The pink spectrum corresponds to the absorption of Cu^{II} alone.

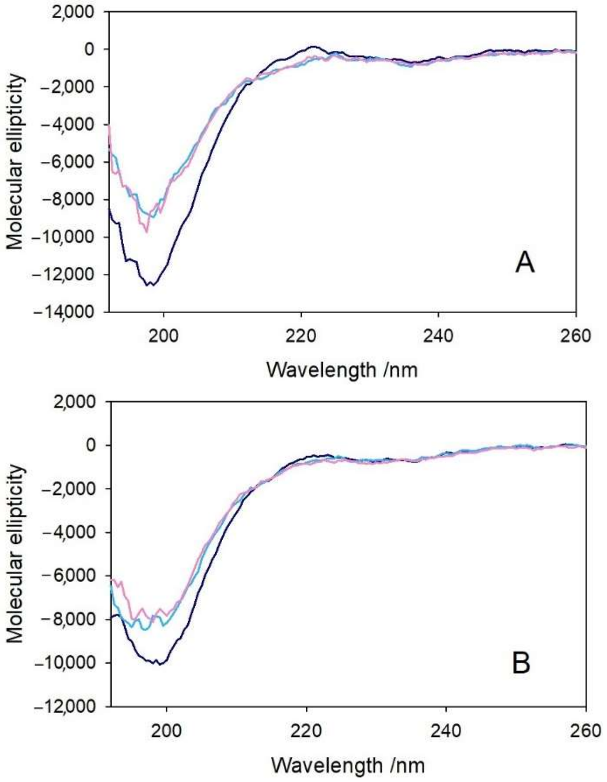

Figure S13. Far-UV CD spectra of 1.1 equiv. A$\beta_{1\text{-}16}$ peptide (10 μM, panel A) and 1.1 equiv. A$\beta_{1\text{-}28}$ peptide (5.5 μM, panel B) in 5 mM phosphate buffer solution at pH 7.4 (blue traces) and upon the addition of copper(II) (1 equiv., light blue) and of dopamine (1 equiv., pink)

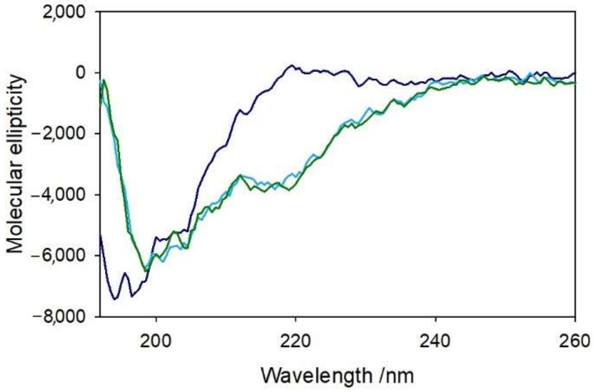

Figure S14. Far-UV CD spectra of 1.1 equiv. A$\beta_{4\text{-}16}$ peptide (10 μM) in 5 mM phosphate buffer solution at pH 7.4 (blue traces) and upon the addition of copper(II) (1 equiv., light blue) and after 15 minutes of incubation (green).

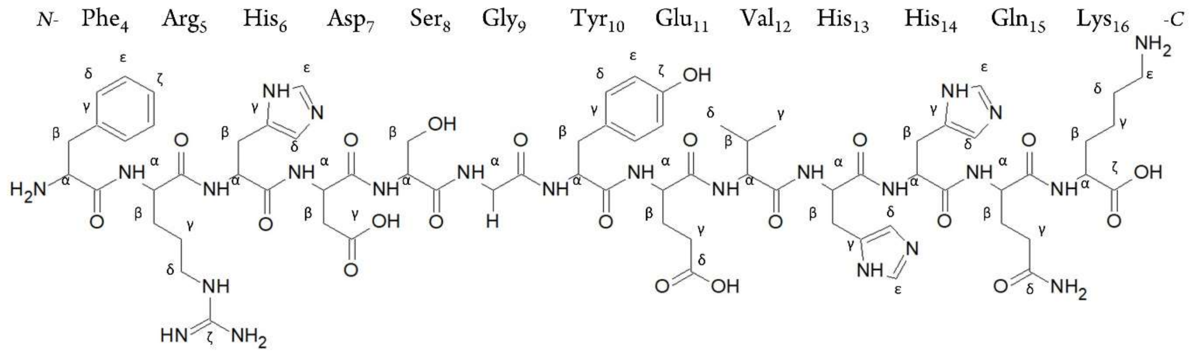

Chart S1. Representation of the amino acidic sequence of Aβ4-16 peptide with the identifications of protons in each residue.

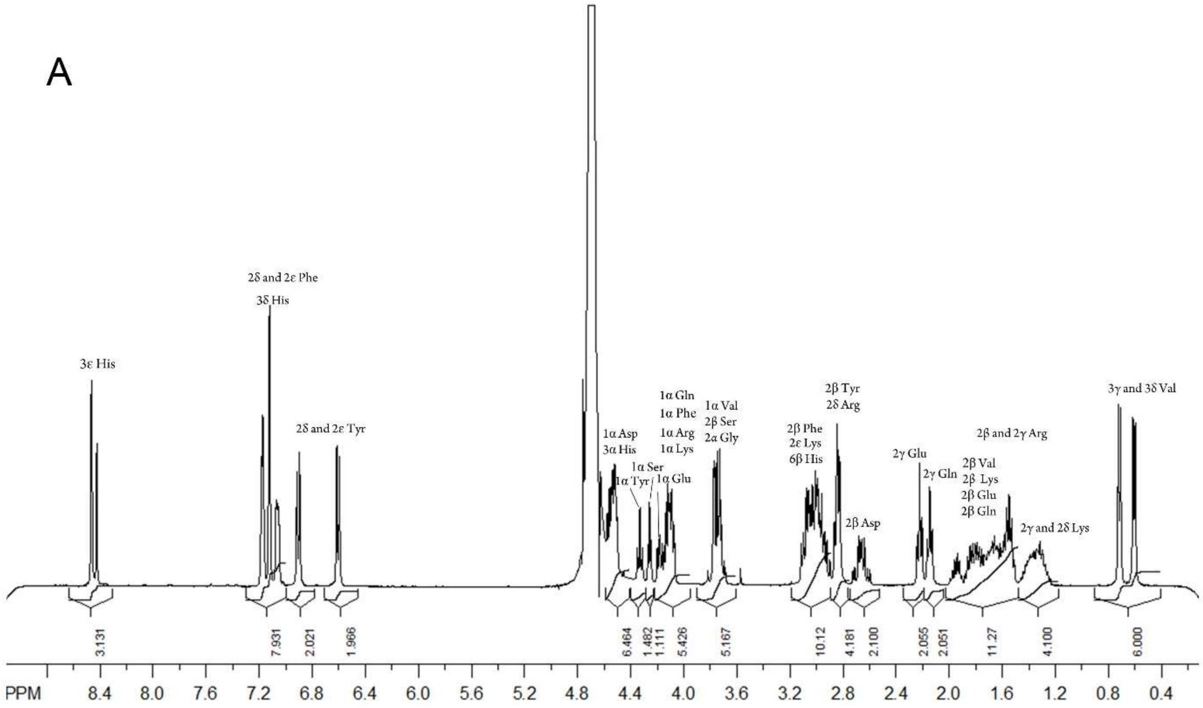

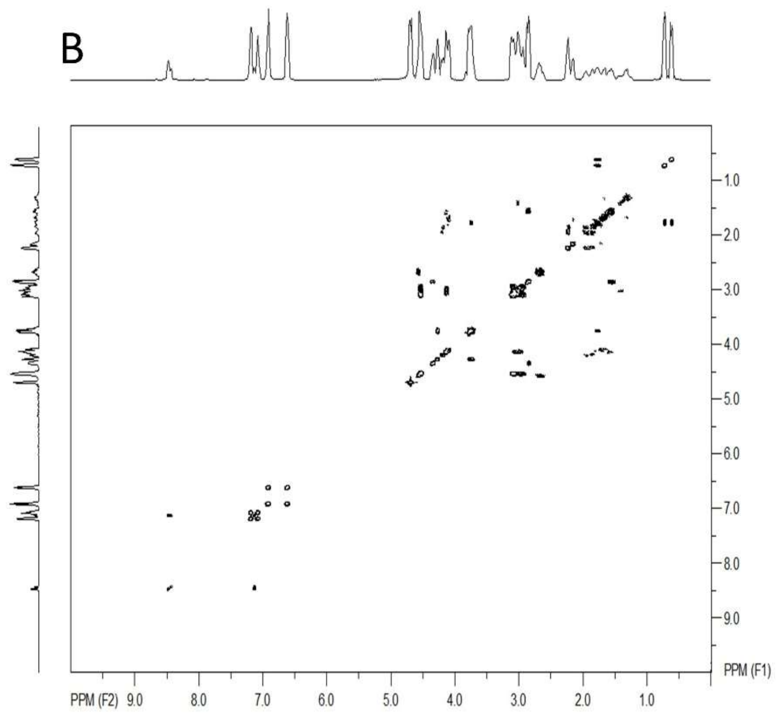

Figure S15. ^{1}H-NMR spectrum (panel A) of A$\beta_{4\text{-}16}$ peptide (8.6 mM) in phosphate buffer solution at pH 7.4 with the proton assignment of each residue. ^{1}H-COSY (panel B) performed on the same sample was used to assign the signals.

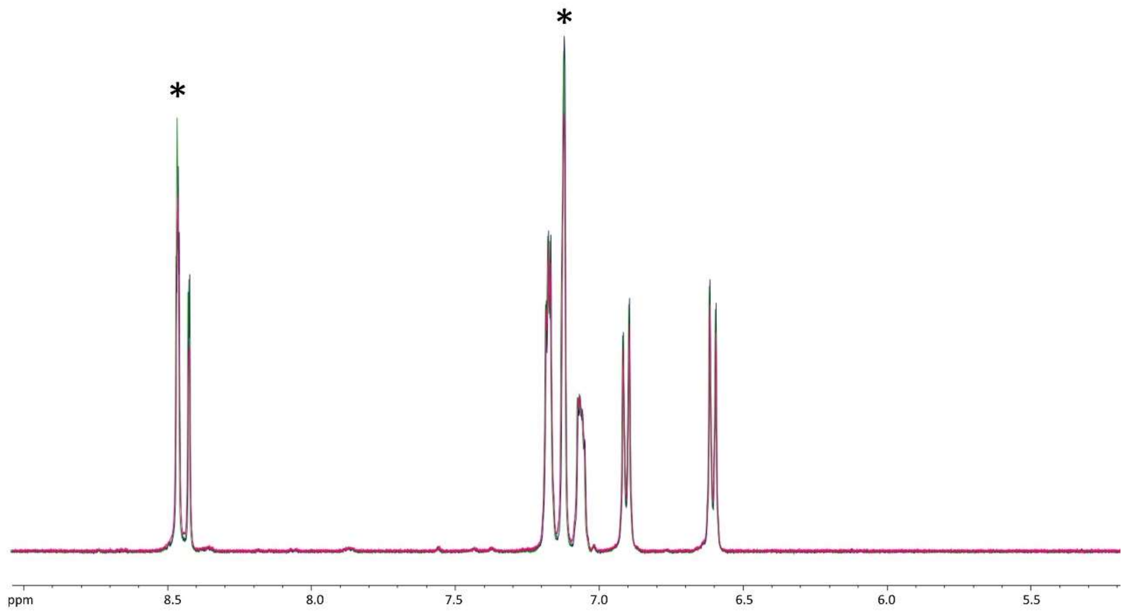

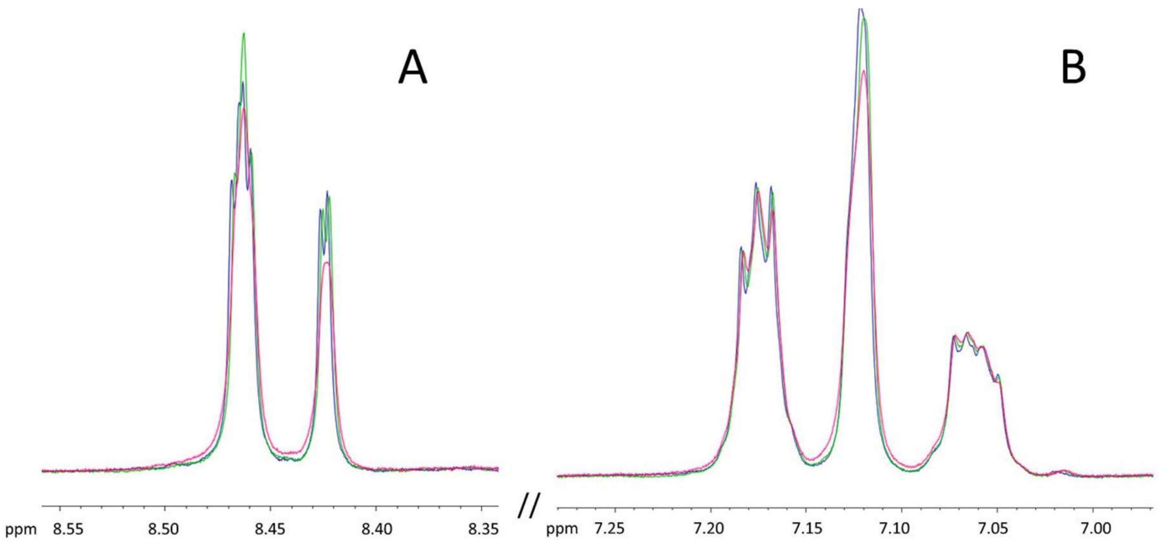

Figure S16. ^{1}H-NMR spectrum in the aromatic region of A$\beta_{4\text{-}16}$ peptide (8.6 mM, 1.1 equiv., blue spectrum) in phosphate buffer solution at pH 7.4 with the addition of 1 equiv. Ni^{II} (green spectrum) and 1/100 equiv. Cu^{II} (pink spectrum). Panel A and B show the peaks more affected by the presence of metals, corresponding to the His signals.

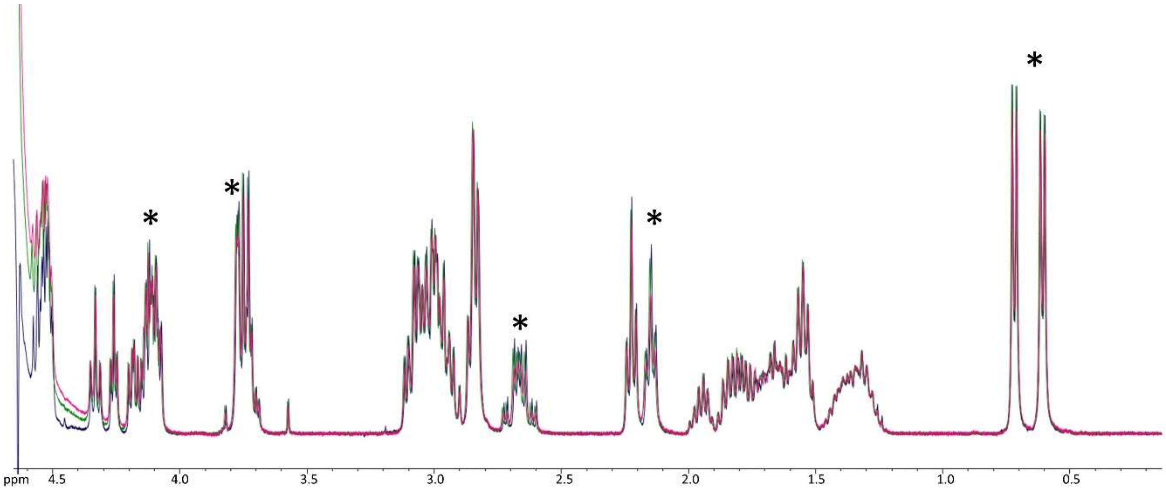

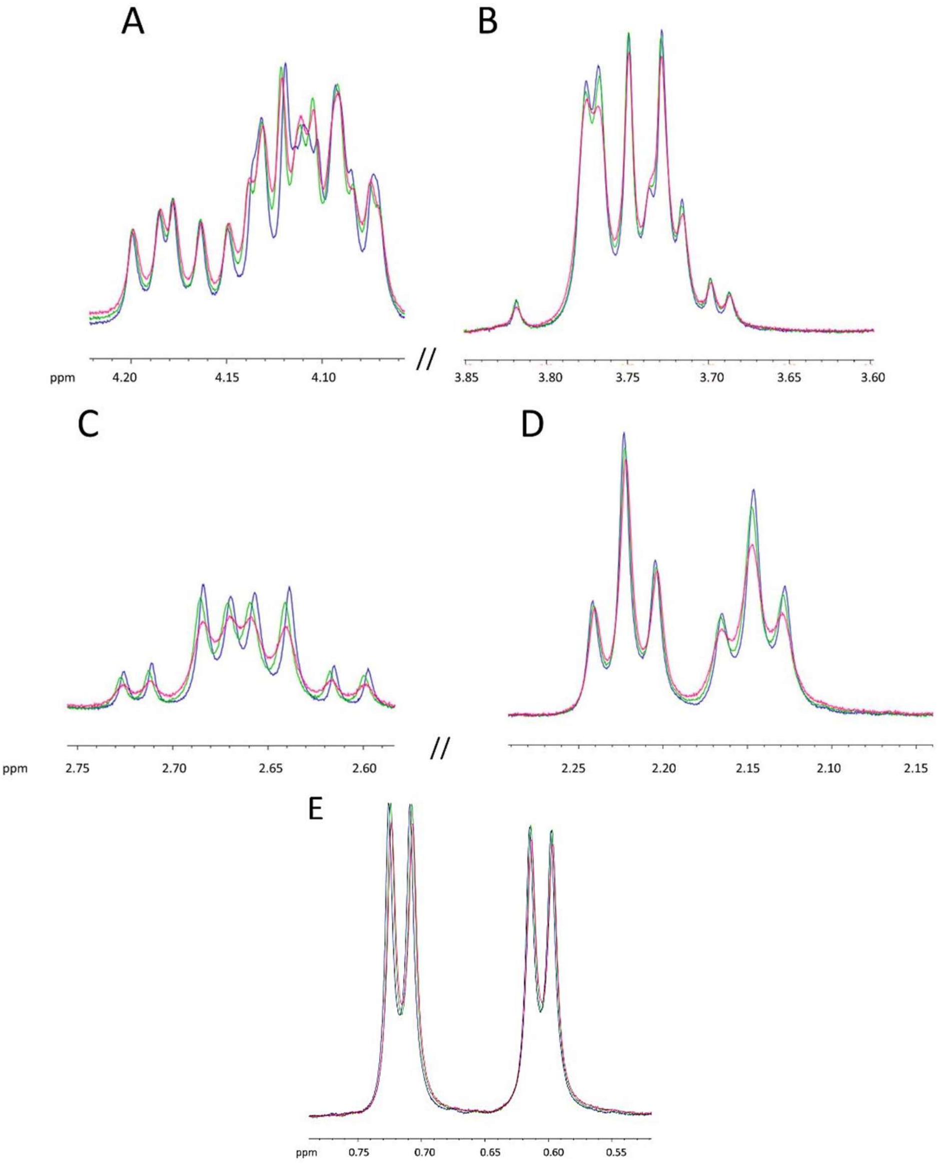

Figure S17. ^{1}H-NMR spectrum in the aliphatic region of Aβ$_{4\text{-}16}$ peptide (8.6 mM, 1.1 equiv., blue spectrum) in phosphate buffer solution at pH 7.4 with the addition of 1 equiv. Ni^{II} (green spectrum) and 1/100 equiv. Cu^{II} (pink spectrum). Panel A, B, C, D and E show the peaks more affected by the presence of metals.

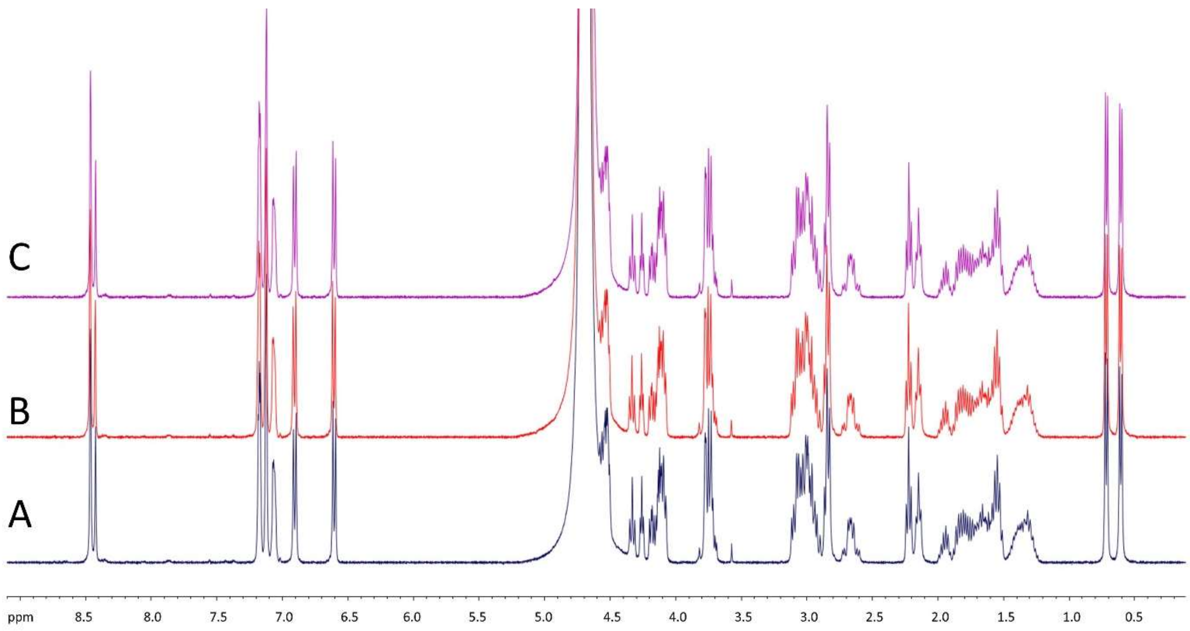

Figure S18. ^{1}H-NMR spectrum of [Ni-Cu-A$\beta_{4\text{-}16}$] adduct at 1:0.01:1.1 molar ratio in phosphate buffer solution at pH 7.4 (spectrum A) and upon the incubation of 6 (spectrum B) and 12 hours (spectrum C).
